# The effect of exogenous melatonin on reducing scoliotic curvature and improving bone quality in melatonin-deficient C57BL/6J mice

**DOI:** 10.1038/s41598-019-42467-5

**Published:** 2019-04-17

**Authors:** Hao Liu, Zhen Liu, Chi-wai Man, Jing Guo, Xiao Han, Zongshan Hu, Tzi Bun Ng, Zhihui Zhao, Jie Li, Weijun Wang, Tseng-chang Chun, Jun Qiao, Benlong Shi, Leilei Xu, Hongda Bao, Qing Jiang, Tsz Ping Lam, Jack Chun Yiu Cheng, Yong Qiu, Zezhang Zhu

**Affiliations:** 1 Department of Spine Surgery, The Affiliated Drum Tower Hospital of Nanjing University Medical School, Nanjing University, Nanjing, China; 2 SH Ho Scoliosis Research Laboratory, Department of Orthopaedics and Traumatology, Prince of Wales Hospital, The Chinese University of Hong Kong, Shatin, NT, Hong Kong SAR, China; 3 School of Biomedical Sciences, The Chinese University of Hong Kong, Shatin, NT, Hong Kong SAR, China; 4 Joint Scoliosis Research Center of the Chinese University of Hong Kong & Nanjing University, The Chinese University of Hong Kong, Shatin, NT, Hong Kong SAR, China; 5 Spine Surgery, The First Affiliated Hospital of Guangzhou Medical University, Guangzhou, China; 6 Department of Sports Medicine and Adult Reconstructive Surgery, The Affiliated Drum Tower Hospital of Nanjing University Medical School, Nanjing University, Nanjing, China; 7 Department of Neurosurgery, The Affiliated Drum Tower Hospital of Nanjing University Medical School, Nanjing University, Nanjing, China

**Keywords:** Experimental models of disease, Risk factors

## Abstract

It is well-documented that melatonin deficiency has been linked to the etiopathogenesis of adolescent idiopathic scoliosis. In this study, we intended to apply melatonin in melatonin-deficient mice to ascertain whether melatonin could reduce the incidence/severity of scoliosis, and investigate the role of melatonin on bone mineral density in scoliosis. A total of 80 mice were divided into 4 groups: 20 quadrupedal mice and 20 bipedal mice served as controls; 20 quadrupedal and 20 bipedal mice received oral melatonin (8 mg/kg BW) daily. After 5^th^, 10^th^, 15^th^ and 20^th^ weeks of treatment, radiographs and *in vivo* micro-CT were used to determine the incidence of scoliosis and bone qualities, respectively. Upon sacrifice, the levels of melatonin were measured in each group. At 20^th^ week, the occurrence of scoliosis was 80%, 30%, 22% and 5% in bipedal, quadrupedal, bipedal + melatonin and quadrupedal + melatonin group, respectively. The trabecular bone quality of the vertebral body was significantly ameliorated in the melatonin-treated bipedal models. Likewise, the number of osteoclasts was significantly less in those treated with melatonin. Our results indicated that melatonin deficiency may be crucial for scoliotic development, and restoration of melatonin levels can prevent scoliotic development with the improvement in bone density.

## Introduction

Adolescent idiopathic scoliosis (AIS) is a three-dimensional deformity of the spine which occurs mainly in girls between the ages of 10 to 18^1^. Previous studies have shown that AIS is associated with generalized low bone mineral density (BMD) that can persist beyond skeletal maturity^2–5^. However, despite the well-known clinical manifestations of AIS, its pathogenesis and relationship to low BMD remain unclear^6,7^.

An association between osteopenia and acquired spine deformity in AIS was first reported in the early 1980’s^2^. A similar study disclosed that AIS subjects exhibited significantly lower lumbar spine and proximal femur BMDs compared with age-matched control subjects^3^. And a longitudinal follow-up on these patients demonstrated a persistently low BMD status^8^. In addition, our team was the first to demonstrate that low BMD or osteopenia could serve as a significant prognostic factor for predicting curve progression in AIS with an adjusted odds ratio of 2.3^9^.

Besides a diminished bone density, abnormal bone qualities were also noted in AIS patients. Our previous study showed a contracted bone volume fraction and trabecular thickness with low osteocyte and osteoblast counts in AIS^10^. Recently, our study using quantitative ultrasound (QUS) also revealed significantly lower broadband ultrasound attenuation (BUA) and stiffness index (SI),lending support to the presence of a deteriorated bone quality in AIS^11^. By utilizing high-resolution peripheral quantitative computed tomography (HR-pQCT), it demonstrated a malformation in cortical bone parameter (i.e. reduced cortical bone area and cortical bone volumetric BMD) and trabecular bone parameters (i.e. reduced trabecular number and increase in trabecular separation) in patients with AIS^12^. Interestingly, the alteration of these bone qualities in osteopenic AIS was worse than in osteopenic controls, particularly in the trabecular bone compartment. In addition, our team has reported abnormal proliferation and differentiation of osteoblasts in AIS girls in response to exogenous melatonin^13,14^.

Throughout the past decades, many scoliotic animal models have been established to unravel the etiology of AIS^15–18^. The pinealectomized chicken is the most widely used experimental model for exploring the etiology of AIS^19^. With the ablation of the pineal gland, it would result in a significant declined in serum melatonin level, which ultimately will bring about scoliotic curvature in chickens^20–22^. However, this invasive surgical technique has been criticized to cause extensive damage to adjacent parts of the brain, which might in turn lead to some artifacts^9^. Additionally, the incidence of scoliosis in pinealectomized chickens remains controversial (from 48–100%)^23–25^. Thus, in 2006, Machida *et al*.^17^ introduced bipedal ambulation of the melatonin-deficient C57BL/6J mice as a new experimental model for AIS research. An advantage of this model is that this murine strain produces little or no plasma and pineal melatonin due to a natural knock-down of the gene encoding serotonin N-acetyltransferase in dispensable for melatonin biosynthesis^26^. Hence pinealectomy is not required. Likewise, many studies have shown a naturally occurrence of low BMD in C57BL/6J mice when compared with various inbred mouse species. Thus, this trait render an ideal candidates for assessing the effect of melatonin on bone formation *in vivo*. In addition, mice are phylogenetically and physiologically closer to humans than chickens, making it logically and scientifically more relevant as a scoliotic model for AIS research^17,27,28^. Although it has been established that this bipedal melatonin-deficient mouse model develops scoliosis, little is known about the bone micro-architecture in this model. The aim of this study is to investigate the micro-structural properties of the vertebrae and demonstrate the effects of exogenous melatonin on the abnormal bone density and qualities in the bipedal C57BL/6J mouse model.

## Results

### Physiological markers

**Figure 1:**
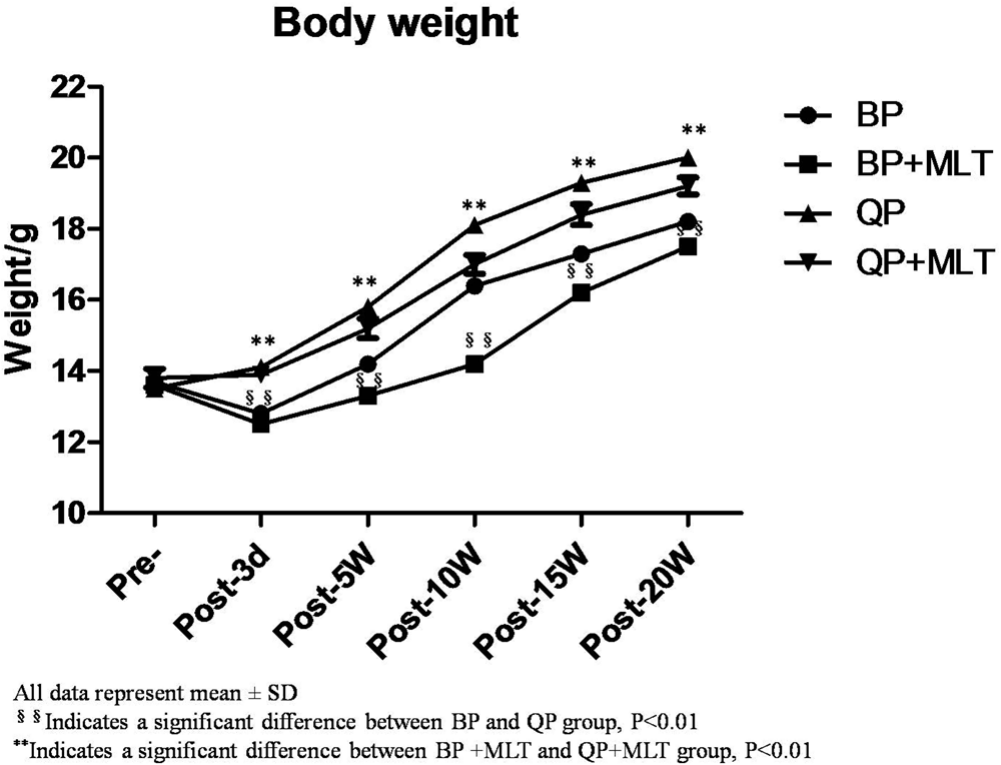
Changes in body weight in the four groups of mice throughout the course of the experiment. The differences in body weight of the bipedal mice (BP) (n = 20), quadrupedal mice (QP) (n = 20), bipedal (BP + MLT) (n = 18) and quadrupedal mice (QP + MLT) (n = 19) throughout the 5 months.

The changes in body weight after bipedal ambulation were recorded (Fig. 1). Body weights in the BP group declined after operation compared to their weights at entry, without statistically significant difference between the four groups perioperatively (p > 0.05). Five weeks later, mice in both bipedal group and bipedal + melatonin group exhibited a gradually increasing body weight. Mice in the bipedal + melatonin group weighed significantly less than the bipedal group (14.2 ± 1.26 vs.16.4 ± 2.09, p < 0.05), during the first 10 weeks postoperatively and at 20 weeks the difference was no longer significant (18.2 ± 2.12 vs. 17.5 ± 2.63, p > 0.05). Two mice in the bipedal + melatonin group and one mouse in the quadrupedal + melatonin group were sacrificed owing to complications (e.g. anorexia).

### Biochemical melatonin concentration in serum

The results showed that within each experimental group, the average serum melatonin levels of mice receiving melatonin treatment (193.6 ± 78.4 pg/ml in bipedal + melatonin group and 219.2 ± 65.9 pg/ml in quadrupedal + melatonin group) were significantly higher than those of the bipedal and quadrupedal mice without melatonin treatment, respectively (6.4 ± 4.7 pg/ml in bipedal group and 4.9 ± 3.1 pg/ml in quadrupedal group) (P < 0.01) (Table 1).

**Table 1:** Distribution of curve incidences and serum melatonin level among the four groups at 20th week.

Groups	BP (Group 1)	BP + MLT (Group 2)	QP (Group 3)	QP + MLT (Group 4)
N	20	18	20	19
Peripheral serum MT level (pg/ml)	6.4 ± 4.7^§§^	193.6 ± 78.4^**^	4.9 ± 3.1^※※^	219.2 ± 65.9^‡‡^

All data represent mean ± SD.

^§§^Indicates a significant difference between BP and QP + MLT group, P < 0.01.

^**^Indicates a significant difference between BP and BP + MLT group, P < 0.01.

^※※^Indicates a significant difference between QP and BP + MLT group, P < 0.01.

^‡‡^Indicates a significant difference between QPand QP + MLT group, P < 0.01.

### Radiographic measurements of the spinal deformity

**Figure 2:**
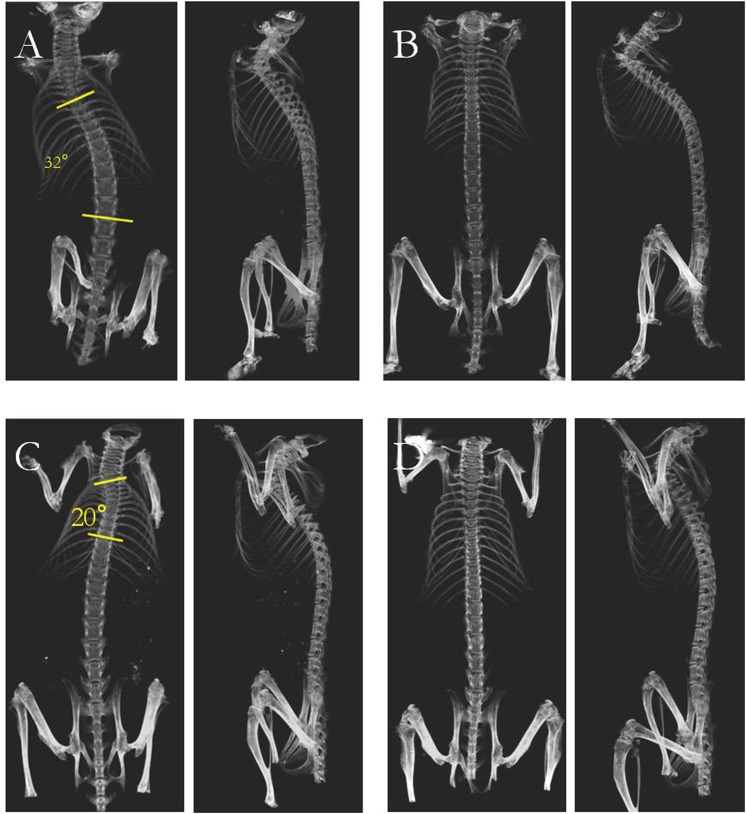
3D characteristics of spine in each group. (**A**) Severe scoliosis in a bipedal mouse (n = 20). It clearly showed scoliosis with right convexity and dissymmetry of thoracic cage. (**B**) A straight spine without vertebral rotation in a bipedal mouse after melatonin treatment (n = 18). It showed straight spine and symmetry of thoracic cage. (**C**) Mild scoliosis of spine in quadrupedal group (n = 20). Helical 3D-CT showed mild asymmetry of thoracic cage. (**D**) A straight spine without vertebral rotation in a quadrupedal mouse after melatonin treatment (n = 19).

**Figure 3:**
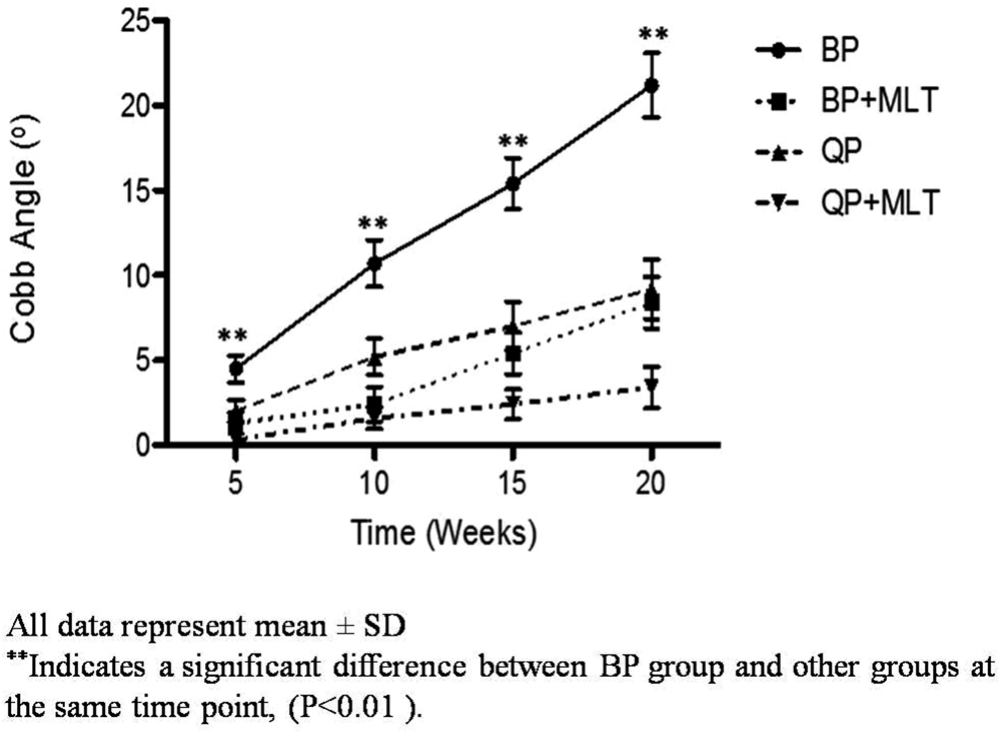
Changes in Magnitude of Cobb Angle throughout the course of the experiment in the 4 groups.

Representative radiographs of a straight spine and a scoliotic spine are shown in Fig. 2. The incidence of scoliosis and Cobb angle of each group at 5, 10, 15 and 20 weeks are shown in Table 2 and Fig. 3, respectively. Scoliosis developed in a larger percentage of animals as time went on: 10% of the animals (2/20; thoracic:2; Cobb angle: 4.5° ± 3.6) at 5 weeks, 50% of the animals (10/20; thoracic:3; thoracolumbar:7; Cobb angle: 10.7° ± 6.2) at 10 weeks, 80% of the animals (16/20; thoracic:6; thoracolumbar:10; Cobb angle: 15.4° ± 6.7) at 15 weeks, and 90% of the animals (18/20; thoracic:7; thoracolumbar:11; Cobb angle: 21.2° ± 8.5) at 20 weeks in the bipedal group (Table 2, Fig. 3). In the wild type control, 70% of the mice in the quadrupedal group (14 out of 20 mice) had a straight spine and symmetry of thoracic cage when examined at 20 weeks (Fig. 3). Only six mice in the quadrupedal group developed scoliosis (thoracic:2;thoracolumbar:4). The magnitude of scoliosis in bipedal group was significantly larger than that in other groups (P < 0.01) (Fig. 3). In the melatonin-treated groups, 4 out of the 18 mice (thoracic:2; thoracolumbar:2) in the bipedal group developed scoliosis whereas only 1 of the 19 mice (thoracolumbar:1) in the quadrupedal + melatonin group developed scoliosis at 20 weeks (Table 2, Fig. 3). In general, the magnitudes of scoliosis in the melatonin-treated groups were smaller than those in groups without melatonin treatment.

**Table 2:** Incidence of scoliosis in the four groups at 20th week.

Groups	BP (Group 1)	BP + MLT (Group 2)	QP (Group 3)	QP + MLT (Group 4)
5 W	10% (2/20)	5% (1/20)	5% (1/20)	0% (0/20)
	T:2	T:1	TL:1	
10 W	50% (10/20)	10%^*^ (2/20)	25% (5/20)	5% (1/20)
	T:3 TL:7	T:1 TL:1	T:2 TL:3	TL:1
15 W	80% (16/20)	21.1%** (4/19)	30%^§§^ (6/20)	5.3% (1/19)
	T:6 TL:10	T:2 TL:2	T:2 TL:4	TL:1
20 W	90% (18/20)	22.2%** (4/18)	35%^§§^ (6/20)	5.3% (1/19)
	T:7 TL:11	T2: TL:2	T:2 TL:4	TL:1

T: thoracic TL: thoracolumbar.

^*^Indicates a significant difference between BP and BP + MLT group, P < 0.05.

^**^Indicates a significant difference between BP and BP + MLT group, P < 0.01.

^§§^Indicates a significant difference between BP and QP group, P < 0.01.

### Micro CT analysis

**Figure 4:**
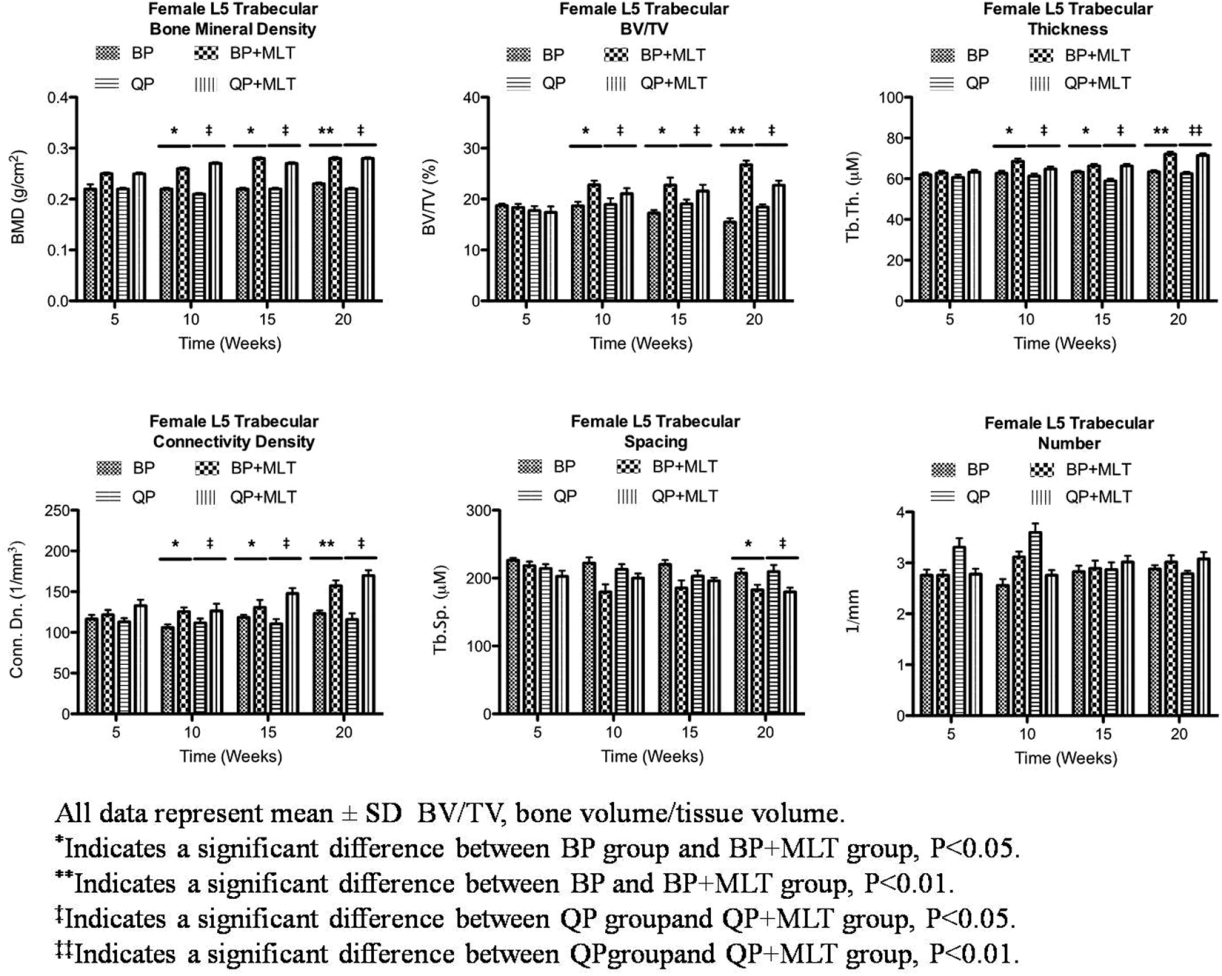
Micro-CT analysis of Trabecular bone architecture.

**Figure 5:**
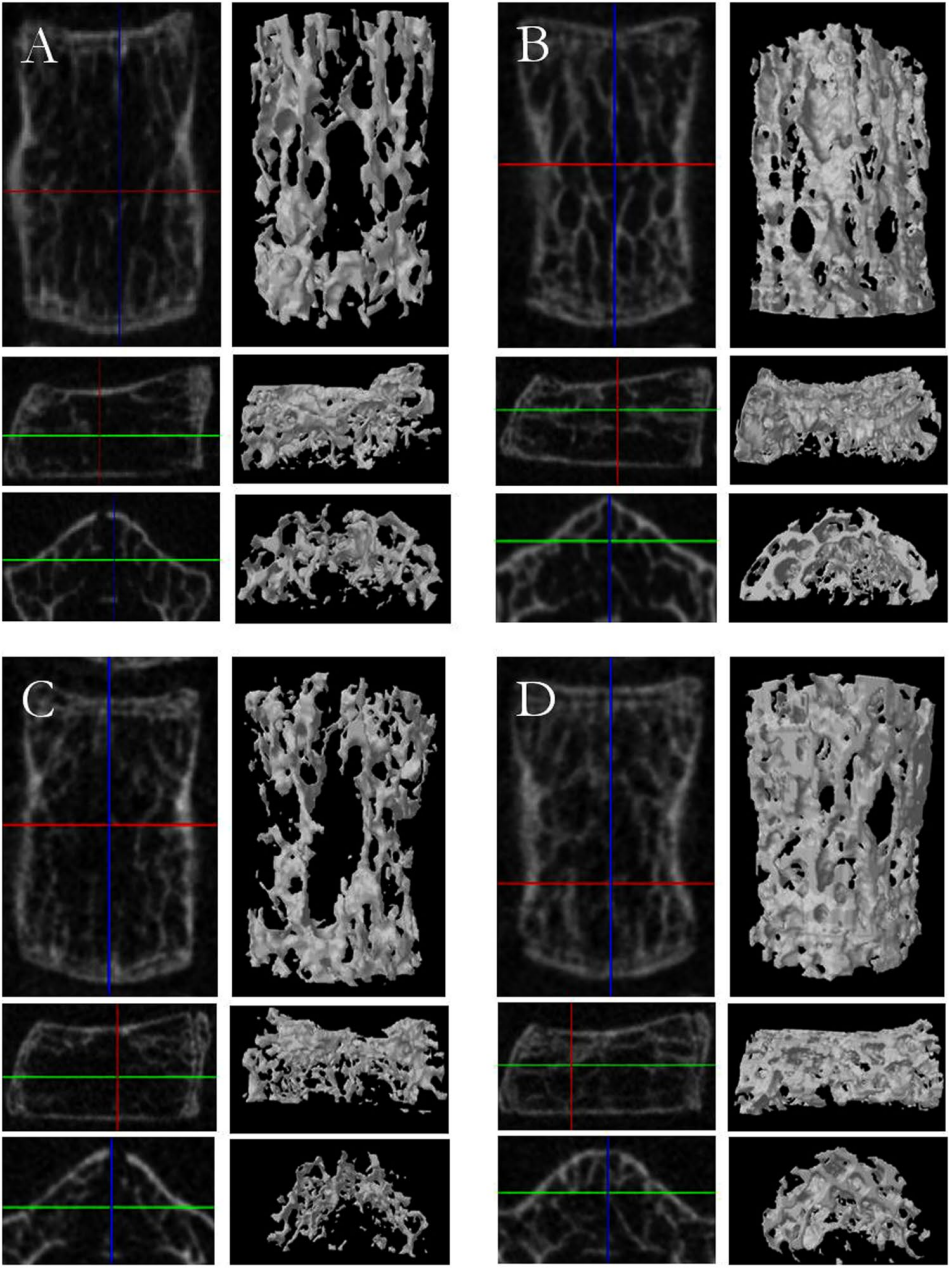
Representative *in vivo* CT images of the fifth lumbar vertebra in each group. (**A**) Bipedal mouse (n = 20), (**B**) bipedal mouse with melatonin treatment (n = 18), (**C**) quadrupedal mouse (n = 20) and (**D**) quadrupedal mouse with melatonin treatment (n = 19). The cancellous bone from bipedal mouse (**A**) and quadrupedal mouse (**C**) without melatonin treatmentshowed rod-like trabeculae with disconnected irregular surfaces, while cancellous bone frombipedal mouse and quadrupedal mousetreated with melatonin (**B,D**) had plate-like trabeculae that were markedly increased, thickened and interconnected.

The 3D measurements of trabecular bone microarchitecture for each group during the 20 weeks changes are shown in Fig. 4. After the initial 5 weeks of treatment, there were no significant differences in the trabecular bone microarchitecture among the four groups. However, the mean value of bone mineral density (BMD) at the fifth lumbar vertebral body of non-melatonin treated groups (bipedal and quadrupedal) began to be significantly lower when compared with the melatonin-treated groups (bipedal + melatonin and quadrupedal + melatonin) after 10 weeks of treatment (P < 0.01). The ratio of bone volume (BV/TV), trabecular thickness (Tb.Th), and connectivity density (Conn.Dn) in the groups without melatonin treatment (bipedal and quadrupedal) were all significantly depressed in comparison with those in the melatonin-treated groups (bipedal + melatonin and quadrupedal + melatonin) after 10 weeks (P < 0.01). Trabecular spacing (Tb,Sp) in the groups without melatonin treatment (bipedal and quadrupedal) was significantly wider than that in the melatonin-treated groups (bipedal + melatonin and quadrupedal + melatonin) at 20 weeks (P < 0.05). However, no significant difference in trabecular number (Tb.N) was found among the groups during the 20 weeks of treatment (Fig. 4). Representative images of the 3D trabecular microstructure of cancellous bone in the fifth lumbar vertebra from each group are presented in Fig. 5. Cancellous bone from groups without melatonin treatment (bipedal and quadrupedal) displayed rod-like trabeculae with disconnected irregular surfaces, while cancellous bone from melatonin-treated groups (bipedal + melatonin and quadrupedal + melatonin) manifested plate-like trabeculae that were markedly increased in number, thickened and interconnected.

### Osteoclast numbers

**Figure 6:**
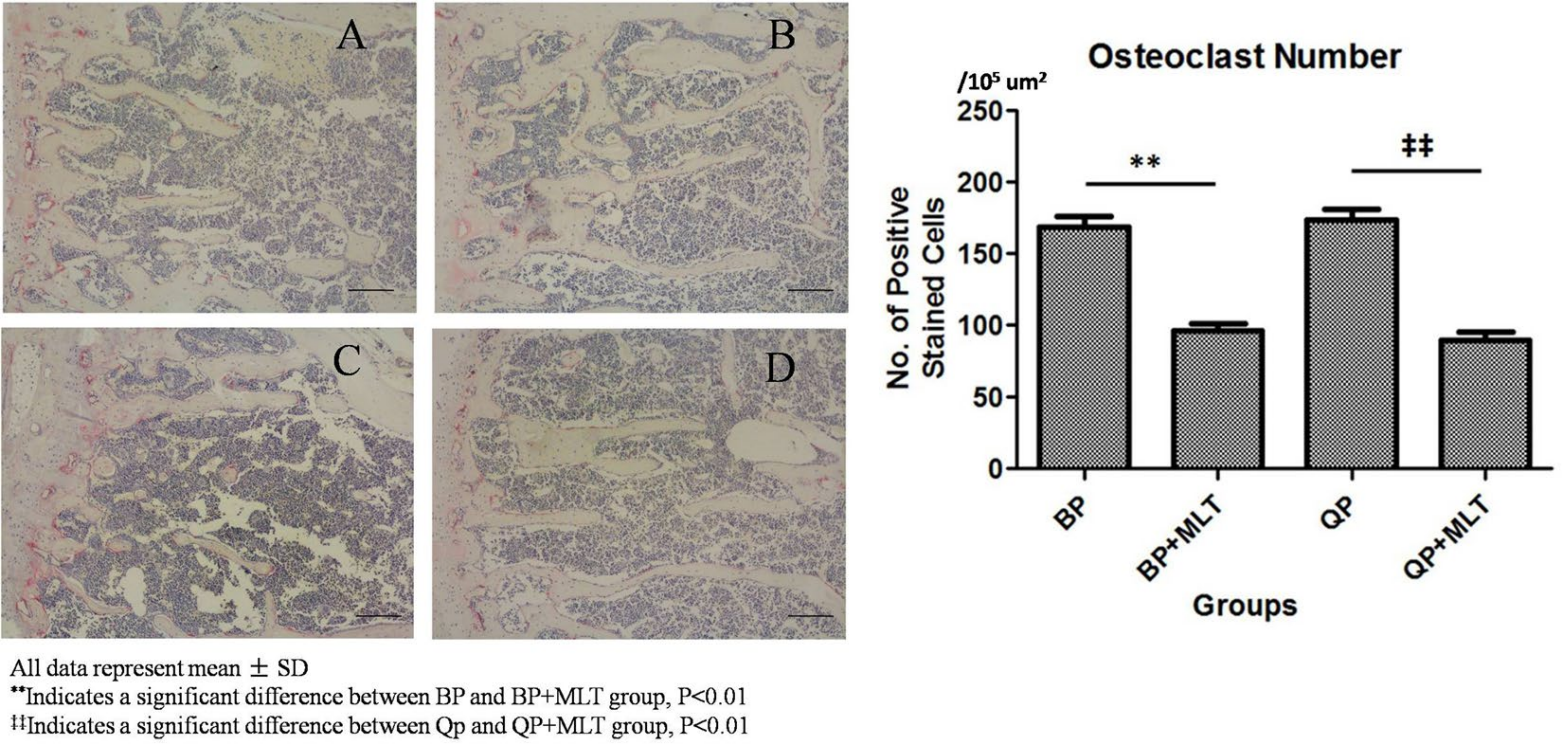
The number of osteoclasts in each group. Note the decreased number of osteoclasts in the BP + MLT and QP + MLT group (**B**,**D**) compared with the BP and QP goup (**A**,**C**). The osteoclasts were stained red. The form and alignment of the chondrocytes were uniform (TRAP stain, Scale bars, 100 um).

Osteoclasts were detected across the entire vertebra by TRAP staining. However, osteoclasts were found to be located mainly at the edge of the growth plate, and were less abundant in the primary and secondary spongiosae. The osteoclast numbers in bipedal group and quadrupedal group were 169.10 ± 30.16/10^5^ um^2^ and 174.05 ± 31.79/10^5^ um^2^, respectively. There were significantly fewer osteoclasts in the bipedal + melatonin group (96.25 ± 21.16/10^5^ um^2^) and quadrupedal + melatonin group (89.85 ± 22.62/10^5^ um^2^) than those in the bipedal group and quadrupedal group, respectively (P < 0.01) (Fig. 6).

## Discussion

In the current study, 3D structural scoliosis developed in 18 out of 20controlbipedal mice and in 6 out of 20controlquadrupedal mice, whereas only 4 out of 18 melatonin-treated bipedal mice and 1 out of 19 melatonin- treated quadrupedal mice developed scoliosis at 20 weeks. In Machida’ study, none of the 30 bipedal mice receiving melatonin treatment (8 mg/kg BW/day) developed scoliosis, whereas 29 out of 30 (97%) bipedal mice without receiving melatonin treatment developed scoliosis. These findings suggest that reduced levels of melatonin in bipedal mice could play a crucial role in the development of scoliosis, and the restoration of the melatonin levels by melatonin treatment prevented the development of scoliosis.

Turgut *et al*.^29^ investigated the histological and radiological changes in the chicken cervical vertebrae following pinealectomy and observed that all pinealectomized chickens developed scoliotic deformity and exhibited a significantly attenuated BMD than their counterparts with an intact Pineal. In the cervical vertebrae, the total number of osteocytes, but not the osteoblast count, was significantly abated in the pinealectomized group compared to the group with an intact pineal but there was no significant difference in the total number of osteoblasts between the two groups. The effects of melatonin on osteogenesis observed histologically were consistent with the results of radiological evaluation and BMD. Thus, it was concluded that melatonin may have an osteo-inductive effect on bone formation. In Hitoshi’s study^30^, BMD in pinealectomized chickens was also significantly lowered than the non-pinealectomized group. However, there was no significant difference in the number of osteoclasts among all groups. In the current study, the number of osteoclasts was significantly lessened in C57BL/6J bipedal mice after melatonin administration. It is generally considered that the osteoclast number has a close association with bone modeling. Bone remodeling occurs in response to changing environmental influences that are associated with aging, menopausal status, lifestyle, and, more recently, melatonin secretion. Even though the data are limited, a role of melatonin in regulating osteoclast activity is emerging. For instance, the decrement in melatonin level is associated with an increase in bone resorption, which suggests that melatonin may act as an endogenous inhibitor of osteoclast activity^31^. In another study, melatonin-mediated inhibition of bone resorption was accompanied by a down regulation in receptor activator of nuclear factor κB ligand (RANKL)-induced osteoclastogenesis^32,33^, and diminished induction of NFATc1 transcription factor^34^. The ability of melatonin to inhibit RANKL-mediated osteoclastogenesis was thought to be associated with its action on osteoblasts to induce osteoprotegerin levels^35^, which is supported by studies demonstrating a melatonin-mediated up-regulation of osteoprotegerin and other osteogenesis markers in pre-osteoblastic MC3T3-E1 cells^32,36,37^, and human osteoblasts^38^. Melatonin induced an upregulation of Osterix (osteoblast differentiation transcription factor) expression during osteoblast differentiation by suppressing ubiquitin-proteasome-mediated Osterix breakdown leading to an augmented bone mineralization^39^. The histological and radiological changes and fall in BMD observed in melatonin-deficient bipedal mice in this study corroborates the data on the action of melatonin on osteoclasts and bone formation. As previously reported, melatonin influences bone metabolism by stimulating bone growth and inhibiting osteoclast activity^36,39,40^, and scavenging of damaging free radicals produced by osteoclasts thus preventing bone resorption^36^. These effects of melatonin could be mediated by the ubiquitin ligases SCF(B-TrCP) and Keap-Cul3-Rbx, or result from a suppressive action on proteasomes^41^. However, further work is required to determine whether the alteration in osteoclast number is directly associated with the development of scoliosis.

In experimental osteoporosis, BMD measured by conventional DXA estimates bone volume through a two-dimensional analysis. In the present study, using the three-dimensional evaluation of trabecular microarchitecture in experimental scoliosis, the microstructural changes in bone matrix after melatonin treatment could be evaluated. The scanned vertebrae (L5) displayed a drop in BMD and changes in various parameters of bone quality (BV/TV, Tb.Th, Tb.N, Tb.Sp, and Conn.Dn), as demonstrated by *in vivo* micro-CT. These results indicate that the C57BL/6J bipedal mice without receiving melatonin treatment exhibited a lowered BMD and systemic osteoporosis. The morphological characteristics of bone tissue in C57BL/6J bipedal mice were similar to those of postmenopausal osteoporosis in humans. In this study, the number of osteoclasts in the lumbar vertebrae was significantly lower after melatonin treatment as compared to the bipedal and quadrupedal groups. Hence, the alterations in bone density and bone quality parameters might be attributed to an adjustment in the modulation of bone modeling. The observed histological effects of melatonin on osteoclastic activity were in keeping with the findings from radiological and *in vivo* micro-CT analyses. Although the difference is statistically significant, biological significance needs to be further investigated.

The possible explanation on the abnormal bone quality in AIS might be explained by melatonin receptor mutations. Although there were many studies indicating the polymorphism in melatonin receptor with AIS, we actually first reported this finding in 2011^13^. In that study, we showed an abnormal melatonin receptor 1B expression in osteoblasts from girls with adolescent idiopathic scoliosis. In latter study, it demonstrated lower MT2 expression of human mesenchymal stem cells from patients with adolescent idiopathic scoliosis in response to melatonin^42^. This result to the decrease in ALP activity, GAG synthesis and upregulated the expression of genes involved in osteogenic and chondrogenic differentiation including, ALP, osteopontin, osteocalcin, runt-related transcription factor 2, collagen type II, collagen type X, aggrecan and sex determining region Y-box 9. Although these studies showed the likelihood of MT2 disturbance in AIS, it remains to be known on how it could directly interact on bone density.

Although the bipedal mouse model has been criticized on the grounds that it is not a naturally occurring model, undeniably the structural development of a scoliotic curvature in bipedal mice represents a good model for investigating scoliosis. Another limitation of this study is the lack of cortical bone outcomes (e.g. cortical thickness, cortical BV). Although L5 spine is the typical area for assessing BMD, the addition of the distal sites (i.e. femur) and the thoracic vertebrae might be a helpful validation to the BMD status. Beside the trabecular detection by vivaCT, it would be more ideal to have histology toward osteoblasts. In addition, there is a lack of enough mechanistic data to elucidate how melatonin is protective against scoliosis. Hence, it would be necessary to examine mediators of osteoclastogenesis, including RANKL and osteoprotegerin, since melatonin’s effects on osteoclasts are mediated, in part, through osteoblasts. In addition, the role of MT2 melatonin receptors in osteoblasts would be of interest since this has already been shown to play a role in adolescent idiopathic scoliosis in humans^43^. Thus, further future study is needed to investigate these osteoclasts and osteoblasts markers in *in vivo* and *in vitro* experiments. Despite these limitations, the effect of melatonin on the development of scoliosis in C57BL/6J mice model with an intact pineal was clearly demonstrated in this study. These findings uncover the association between melatonin deficiency with scoliotic development and deterioration in bone qualities.

In conclusion, the present results indicate that melatonin deficiency in bipedal mice plays a crucial role in the development of scoliosis, and restoration of melatonin levels helps to enhance the low bone density, rectify the abnormal bone quality and arrest the development/progression of scoliotic deformity in these mice.

## Materials and Methods

### Experimental animals and treatment

A total of 80 female melatonin-deficient C57BL/6J mice were housed in group cages in a temperature-controlled room (20–22 °C) with controlled lighting conditions (lights on from 06:00 to 18:00 hr). The mice were provided with free access to clean water and standard mouse chow that was devoid of melatonin or serotonin. The mice were then randomly divided into four groups as follows: (i) 20control bipedal mice receiving 10% ethanol/saline, (ii) 20 control quadrupedal mice receiving 10% ethanol/saline, (iii) 20 bipedal mice with melatonin (purchased from sigma-aldrich) treatment and (iv) 20 quadrupedal mice with melatonin treatment (research grade melatonin). Melatonin was administered intra-peritoneally (8 mg/kg body weight in 10% ethanol/saline at 22:00 hr daily^17^.

Bipedal ambulation was performed on the mice at 3 weeks of age with the animals under general anesthesia by employing a diazepam/ketamine mixture (1:1 ratio, ketamine (0.05 g/ml) and diazepam (5 mg/ml)). The bilateral forelimbs and the tail were tied and excised at the humeroscapular and basal levels, respectively. The upper extremities below the shoulder and tail were cut off with scissors. No bleeding followed if the ligatures were secured. Bipedal mice after removal of forelimbs and tail were housed for 20 weeks in custom-made cages. The levels at which the mice could have access to food and water supplies were progressively raised, so that they could maintain a standing posture most of the time. Quadrupedal mice with intact tails were housed in standard cages which discouraged the bipedal posture. Changes in body weight were recorded before the surgery and again every 5 weeks after the surgery. All experiments were conducted in accordance with institutional guidelines, following the protocol previously approved by the Animal Ethical and Welfare Committee (AEWC) of the Affiliated Drum Tower Hospital of Nanjing University Medical School (IRB Ref no.: 2015-02-16 and 2015-01-03) and were carried out in accordance with the Guide for the Care and Use of Laboratory Animals (8th edition, Institute of Laboratory Animal Resources on Life Sciences, National Research Council, National Academy of Sciences, Washington DC).

### Longitudinal follow-up of X-ray films of the whole spine

The spines of all mice were radiologically examined for the presence of scoliosis at every 5 weeks. In brief, the posteroanterior radiograph of the vertebral column of each mouse were taken in a standardized prone position with the head fixed by a thread on a support inclined at an angle of 30° from the horizontal plane of the table to prevent postural scoliosis under anesthesia. In the evaluation of radiographs of the vertebral column, scoliosis was defined as a lateral curvature of the spine exceeding 10° as measured by the Cobb method (Cobb angle is given by the angle formed between the superior end plate of the maximally tilted upper end vertebra and the inferior end plate of the maximally tilted lower end vertebra.). The curves at different locations on the spinal column were identified in accordance with the definitions of Scoliosis Research Society classification committee. The measurements were conducted blindly by three independent orthopedic surgeons (XH, JG and ZSH).

### Longitudinal evaluation of bone architecture by *in vivo* micro-CT

For evaluation of trabecular bone density and microarchitecture, vertebral body L5 was scanned with an *in vivo* micro-CT system (SkyScan 1176, Bruker, Germany) at a voltage of 70 keV in a spatial resolution of 9 μm for three-dimensional assessment of bone density and quality every 5 weeks (The L5 vertebra that was not involved in the curve was chosen for analysis by micro-CT and histology). A region of interest (ROI) for quantitative analysis of trabecular bone was defined, extending from the proximal to the distal end of the vertebrae. Bone mineral density (BMD) is defined as the volumetric density of calcium hydroxyapatite (CaHA) in a biological tissue in a biological tissue in terms of g.cm^−3^. It is calibrated by means of phantoms with known density of CaHA. A global gray threshold value of 65 corresponding to an equivalent density of 0.413 g/cm^3^ of CaHA, was set for all the analysis^44^. Morphometric analysis was performed using CTAn software v.1.13 for the selected ROI of trabecular bone to evaluate the following bone structural parameters: bone mineral density (BMD), ratio of bone volume and total volume (BV/TV), trabecular bone number (Tb.N), trabecular bone thickness (Tb.Th), trabecular bone separation (Tb.Sp) and connectivity density (Conn.D) were collected and analyzed.

### Collection of blood samples

Five months after surgery, blood samples were collected from each group of mice at midnight under a dim red light (640–700 nm, <2 lx) to determine the peak value of serum melatonin level, as previously described^17^. Whole bloods were taken by direct cardiac puncture with a needle under anesthesia. The blood sample were collected in serum coagulant tubes and centrifuged for 20 minutes at 300 rpm at 4 °C. The serum samples prepared were then stored at −80 °C until assay. The serum melatonin concentrations were analyzed with an enzyme-linked immunosorbent assay (ELISA) using a Quantikine (R&D Systems, Minneapolis, MN, USA), according to the manufacturer’s instruction. The lower detection limit was 3.12 pg/mL.

### Histomorphometric study of bone samples

After sacrifice of the mice at 5 months of age with an overdose of anesthesia, the lumbar spines were resected of soft tissues and processed for quantitative histological evaluation. The fifth lumbar vertebra was removed, fixed in 10% neutral-buffered formalin, decalcified in 5% hydrochloric acid, and then embedded in paraffin. Serial section (3 µm) was cut in the sagittal plane, and the sections were then stained for the presence of tartrate-resistant acid phosphatase (TRAP)-positive cells for the presence of the osteoclasts, as previously described^45^. The number of osteoclasts in the entire L5 vertebral body was manually counted under a microscope. This procedure was also performed by the sixth author (Z.H.) who was blinded to the study.

### Statistical analysis

All data were expressed as the average ± standard deviation (SD). Between-group differences were analyzed by one-way analysis of variance (ANOVA) with α-adjusted Bonferroni post hoc test was used. All statistical analyses were performed by Statistical Package for the Social Sciences (SPSS, version 17.0, Chicago, IL, US). A *P* value of < 0.05 was considered statistically significant.

### Ethical approval and informed consent

All experiments were conducted in accordance with institutional guidelines, following the protocol previously approved by the Institutional Animal Care and Use Committee (IACUC) of the university (IRB Ref no.: 2015-02-16 and 2016-01-03).
